# QoL Following Dacryocystorhinostomy: Linguistic Adaptation of Italian Version of GBI

**DOI:** 10.1007/s12070-023-03638-z

**Published:** 2023-03-23

**Authors:** Elena Cantone, Aldo Torrisi, Aurelio D’Ecclesia, Eva Aurora Massimilla, Giovanni Motta, Gaetano Motta

**Affiliations:** 1 Department of Neuroscience, Reproductive and Odontostomatological Sciences-ENT Section, University of Naples Federico II, 80131 Naples, Italy; 2 Head and Neck Department-ENT Section, AOU Federico II, 80131 Naples, Italy; 3 ENT Operative Unit, IRCCS “Casa Sollievo Della Sofferenza”, San Giovanni Rotondo, Italy; 4 Head and Neck Surgery Unit, Department of Mental and Physical Health and Preventive Medicine, Università Degli Studi Della Campania Luigi Vanvitelli, 80131 Naples, Italy

**Keywords:** QoL, GBI, Dacryocystitis, Dacryocystorhinostomies, DCR

## Abstract

The Glasgow Benefit Inventory (GBI) is a generic patient recorded outcome measure assessing the quality of life of patients undergoing ear nose and troth surgery. Although largely used in the clinical practice, it has never been adapted and validated in the Italian language. The aim of the study was to translate the original GBI from English to Italian and to examine its reliability for use in the Italian adult population of patients undergoing endonasal endoscopic dacryocystorhinostomy. After translation and back-translation of the original English we evaluated the reliability of GBI for use in 79 Italian adults undergoing dacryocystorhinostomies. Reliability of GBI-IT was examined by the internal consistency of the scale (using the Cronbach’s alpha coefficient), and by the test–retest analysis. The GBI-IT showed adequate internal consistency (Cronbach’s alpha = 0.85 for the total scale). The total GBI-IT score showed a strong correlation in retests (CCC 0.87). In conclusion, our study showed that the GBI-IT has satisfactory internal consistency and reliability and is equivalent to the original English version. In addition, it can be considered a valuable measure for both clinical and research uses.

## Introduction

A stable tear film is of utmost importance for maintaining optical quality and normal functioning of the eye [1]. Although epiphora is a commonly reported symptom, about 30.47/100,000, there are few reports on its impact on patients’ daily activities and social lives [1, 2]. Symptomatic epiphora can occur if secreted tears do not drain properly and an important cause of epiphora is narrowing or occlusion of the nasolacrimal duct as following dacryocystitis (DC) [1, 2]. DC is a relative common disease with an increasing incidence of gram-negative bacteria and methicillin-resistant *Staphylococcus aureus* [3]. Although DC is not burdened with mortality, it has a strong impact on the quality of life (QoL) of affected individuals [4]. For instance, tearing is socially embarrassing as it mimics the appearance of persistent crying [1, 2]. In addition, epiphora impacts on QoL by causing spattered glasses, blurred vision, and sore skin [2].

Blockage of the nasolacrimal duct could be bypassed by surgically creating an anastomosis between the lacrimal sac and the nasal cavity over the occlusion site, using external dacryocystorhinostomy (DCR) or endoscopic endonasal dacryocystorhinostomy (EE-DCR) [2].

So far, QoL assessment studies have mostly been performed using the Glasgow Benefit Inventory (GBI) for specific lacrimal procedures [1, 4].

The GBI is a generic patient recorded outcome measure that was first reported by Robinson et al. to assess the QoL of patients undergoing ear nose and troth (ENT) surgery [5]. It has gained widespread popularity diseases and it is designed for use only once post-intervention, as a measure of change related to a specific surgical or medical intervention [4, 5].

GBI is a validated questionnaire sensitive to the change in health status due to a surgical procedure and patient orientated in ENT diseases. However, although, it has been largely used in the ENT clinical practice, the GBI questionnaire has never been adapted and validated in the Italian language [2, 4].

The aim of the current study was to translate the original GBI from English to Italian and to examine its reliability for use in the Italian adult population of patients undergoing EE-DCR.

## Materials and Methods

The GBI is a validated questionnaire which can be completed by interview or self-completed by patients [5]. It is composed of 18 questions answered using a five-point Likert scale, addressing change in health status post any intervention [5]. The GBI is divided into three distinct subscales: 12 questions focusing on “general” changes in health status, as well as changes in psychosocial health status defined (general); 3 questions related to the “social support” needed in relation to the pathological condition (social); 3 questions related to the changes in “physical health” including need for medications and number of visits a required doctor (physicists) [5]. The responses are then scaled and averaged to give a score with a range − 100 (poorest outcome) through 0 (no change) to + 100 (best outcome) [5].

**Fig. 1:**
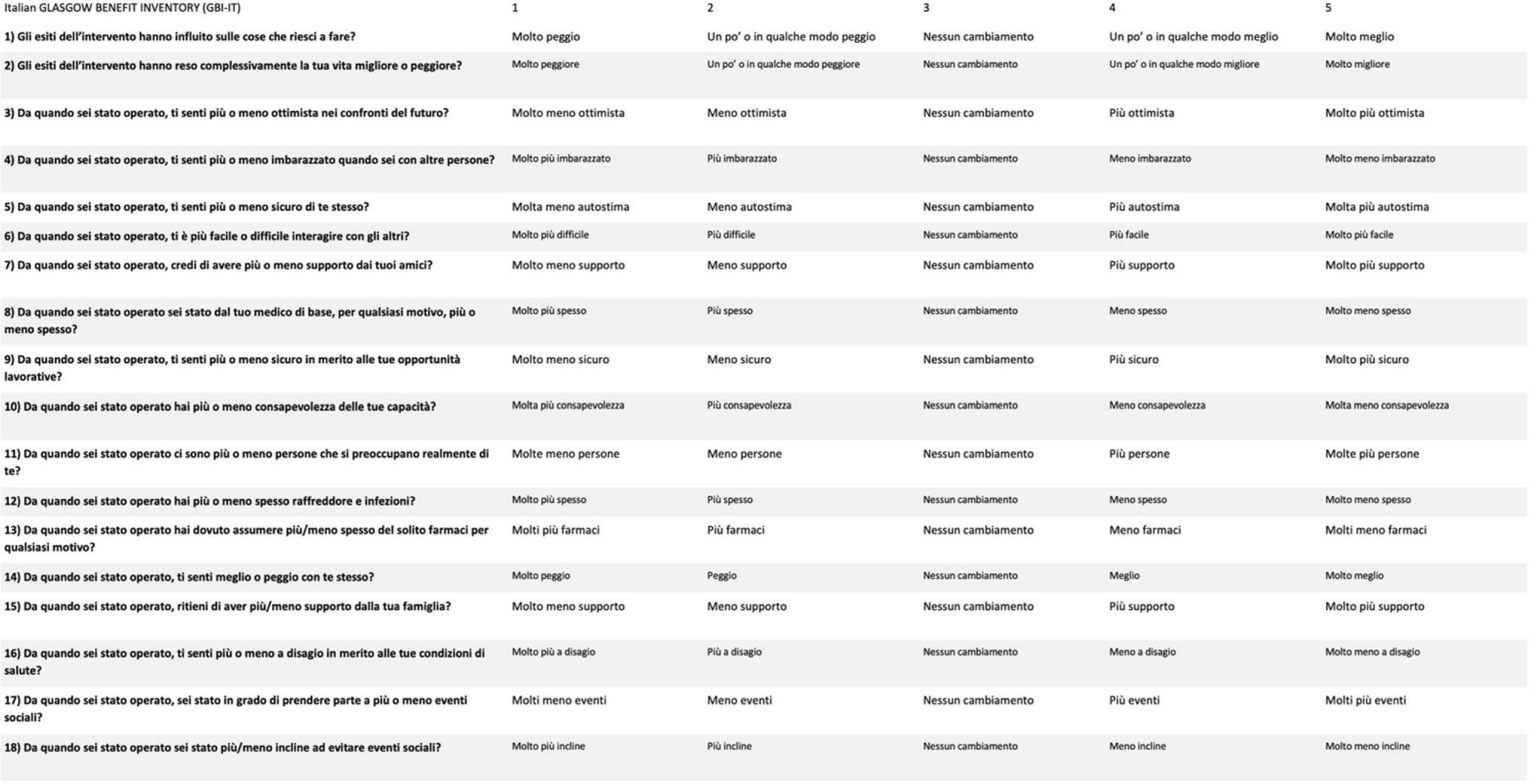
Italian Glasgow Benefit Inventory (GBI-IT)

The original English version of the original GBI questionnaire was first translated into Italian, and the translation was reversed into English, then back into Italian by an independent bilingual Italian native speaker, and two clinicians. The 3 versions were unified by consensus between the translator and the physicians. The final version was translated backwards by an independent bilingual Italian-English speaker with an English mother tongue. After the back-translation, a committee composed of 3 clinicians plus an expert translator, native Italian, adapted the Italian questionnaire to resolve the small discrepancies and provided an Italian version equivalent to the original and understandable version. In order to assess any difficulties in understanding the questionnaire, the Italian version of the GBI was first tested on a sample of 20 DC volunteers. After some aspects were modified to facilitate understanding, the Italian translation was finalized. The Italian GBI questionnaire (GBI-IT) (Fig. 1) was then administered during the follow up to a total population of 79 subjects with DC, who had undergone EE-DCR for a total of 111 eyes (16 bilateral DC) and 30 healthy volunteers. Exclusion criteria were as follows: age under 18, lack of full legal capacity, immunodeficiency, diabetes, oncological, immunological, malformative and neuropsychiatric diseases, inability to read and understand Italian, and complete the questionnaire. Furthermore, the GBI-IT values were compared with the clinical parameter “epiphora” evaluated with the visual analogue scale (VAS). The VAS questionnaire evaluates the severity of a symptom or sign. The staircase consists simply of a 10 cm segment which at the ends has two "end points" defined as "absence of epiphora", which corresponds to "0", and the "worst epiphora that you can imagine" which corresponds to "10". Subjects were asked to mark the degree of severity of the epiphora as perceived at that moment at a point on the scale. The interval between the two extremes is marked every centimeter and allows to attribute value to the subjective disturbance perceived by the patient. Disease was divided into mild, moderate, and severe based on total severity VAS scores (mild, VAS = 0-3; moderate, VAS = 4−7; and severe, VAS = 8–10). At VAS > 5, patient QoL considered unsatisfactory. The study was approved by the local Ethical Committee and patients signed an informed consent.

### Statistical Analysis

The data obtained were analyzed with descriptive statistics and distribution tests as the mean, standard deviation, and Pearson's test. For the tests, *p* < 0.05 were considered statistically significant.

The reliability of GBI-IT was examined using two approaches: the internal consistency of the scale, using Cronbach α coefficient for the total scale and for each subscale; and the test–retest analysis, obtained by asking patients who had completed the GBI-IT questionnaire to complete it again after an interval of four weeks (i.e. an interval of time long enough to ensure that participants would not remember their responses, but short enough to avoid significant changes in their outcome). Test–retest reliability was measured using Lin's Concordance Correlation Coefficient (CCC) and examined the relationship between baseline and four-week-later score. All statistical analyzes were conducted using the R Statistical Platform (vers. 4.0.1).

## Results

The GBI-IT questionnaire was pretested in a pilot study on 20 patients with DC. These patients were selected to represent different ages, sexes, and treatment modalities. After some minor layout changes, no one found the questions difficult to understand, upsetting or disturbing.

For the study we enrolled 79 Caucasian Italian-speaking patients (mean age was 54 ± 14 years, range 35-71; 60 females) diagnosed with DC and undergoing DCR with a total of 111 surgical procedures (32 bilateral DCR) and 30 healthy Italian-speaking Caucasian volunteers (age mean 53± 13 SD, range 24-74; 21 females) over a period from February 2018 to July 2022 (Table 1).

**Table 1:** Population characteristics

No. of patients	79
Procedures	111
Bilaterality	16
Sex	60F
Age	54 ± 14 years
GBI-IT total score	74.86
GBI-IT general	66.7
GBI-IT social	61.21
GBI-IT physicists	62.22
Postoperative VAS epiphora	3.5 ± 1.2

*GBI* Glasgow Benefit Inventory, *VAS* visual analogue scale

All subjects completed the GBI-IT questionnaire at baseline, at least 6 months after surgery, and after 4 weeks for the retest.

The interval time of 4 weeks was not so long that the clinical picture might have changed, but long enough that subjects did not remind previous answers.

The mean total score of the GBI-IT was 74.86 for DC patients, and 97.7 for 30 healthy volunteers (Table 1).

The mean GBI-IT subscales were as follows: general 66.7, social 61.21, physicists 62.22 in DC patients, and general 84.3, social 70.7, physicists 82.21 in healthy volunteers (Table 1).

The GBI-IT mean total scores and the 3 subscales were significantly higher in controls than in patients with DC (*p* <0.05). The mean VAS score for patients with DC was 9.1 ± 0.89 (mean ± SD) before surgery and 3.5± 1.2 after surgery (*p* > 0.05), for healthy people was 0 both before and after surgery (Table 1). We found a significative correlation between postoperative VAS for epiphora and GBI-IT (*p* <0.05).

Internal consistency was supported by a Cronbach α of 0.85 for the total score, and for the different subscales ranged from 0.69 to 0.81. The CCC for the total scale was 0.87, and for the different subscales ranged from 0.71 to 0.88 (Table 2). Age and gender did not affect the evaluation of GBI-IT scores (*p* > 0.05).

**Table 2:** Internal consistency

Reliability	Total score	Range
Cronbach α	0.85	0.69–0.81
CCC	0.87	0.71–0.88

*CCC* concordance correlation coefficient

## Discussion

In the era of precision medicine questionnaires are largely used in the clinical practice to provide quantitative measures of the impact of different medical and surgical treatments on patients' QoL.

Different specific disease symptom questionnaires are available in ENT field, as the sinus-nasal outcome test (SNOT-22) or the chronic otitis media outcome Test 15 (COMOT-15). However, the results of these questionnaires are not comparable with each other. [6–10]

Given the heterogeneous nature of ENT surgeries, it would be desirable to have a universally available questionnaire for all surgical ENT procedures. The GBI is a generic patient recorded outcome measure that has gained widespread popularity in the ENT clinical practice.

The aim of this study was to validate the Italian version of the GBI-IT. According to our results, Cronbach α coefficient for the total scale was 0.85 showing a strong correlation in the retest.

The internal consistency of the GBI-IT was in accordance with several previous studies [11, 12].

The GBI-IT was well accepted by both enrolled patients and healthy volunteers, no one found the questions difficult to understand, upsetting or disturbing. All subjects completed the GBI questionnaire at baseline and at retest after 4 weeks. As expected, the GBI mean total scores and the 3 subscales were significantly higher in controls than in patients with DC.

We also administered the VAS score for epiphora and found a statistically significative correlation between postoperative VAS for epiphora and GBI-IT, demonstrating the reliability of GBI-IT in the assessment of DCR related symptoms.

In the literature there are two questionnaires not translated or validated in Italian, the Lac-Q (Lacrimal symptom questionary) and the NLDO-SS (Nasolacrimal duct obstruction-symptom score), designed to evaluate the success of the DCR intervention [13, 14]. Although they are more specific for the symptoms resulting from obstructive tear pathology, the GBI can evaluate both the symptoms due to the disease and the psychological and social features.

Currently, there has been growing literature exploring questionnaire patient-recorded outcome measures able to assess clinical patient-based outcomes, or rather, outcomes as experienced by the patients. Literature data have demonstrated the benefit of QoL questionnaires [6–9]. Thus, it is of utmost importance developing QoL measures in native languages and appropriate to the culture of a country. In our opinion, a subjective tool, like a questionnaire, can reflect QoL much more closely than exclusive clinician-rated outcomes.

In conclusion, our study showed that the GBI-IT has satisfactory internal consistency and reliability and is equivalent to the original English version. In addition, it can be considered a valuable measure for both clinical and research uses.

## Data Availability

Data are available upon reasonable request.

## References

[1] Shin JH, Kim JD, Woo KI (2015). Korean Society of Ophthalmic Plastic and Reconstructive Surgery (KSOPRS). Impact of epiphora on vision-related quality of life. BMC Ophthalmol.

[2] Jutley G, Karim R, Joharatnam N, Latif S, Lynch T, Oliver JM (2013). Patient satisfaction following endoscopic endonasal dacryocystorhinostomy: a quality of life study. Eye (Lond).

[3] Chung SY, Rafailov L, Turbin RE, Langer PD (2019). The microbiologic profile of dacryocystitis. Orbit.

[4] Hendry J, Chin A, Swan IRC, Akeroyd MA, Browning GG (2016). The Glasgow Benefit Inventory: a systematic review of the use and value of an otorhinolaryngological generic patient-recorded outcome measure. Clin Otolaryngol.

[5] Robinson K, Gatehouse S, Browning GG (1996). Measuring patient benefit from otorhinolaryngological surgery and therapy. Ann Otol Rhinol Laryngol.

[6] Cavaliere M, Capriglione P, Cavaliere F, De Corso E, Zanoletti E, Motta G, Iengo M, Cantone E (2021). Cross-cultural adaptation and Italian validation of chronic otitis media outcome test 15 (COMOT-15). Acta Otorhinolaryngol Ital.

[7] Cantone E, De Corso E, Ricciardiello F, Di Nola C, Grimaldi G, Allocca V, Motta G (2022). Olfaction recovery following dupilumab is independent of nasal polyp reduction in CRSwNP. J Pers Med.

[8] De Corso E, Montuori C, Settimi S, Mele DA, Cantiani A, Corbò M, Cantone E, Paludetti G, Galli J (2022). Efficacy of biologics on refractory eosinophilic otitis media associated with bronchial asthma or severe uncontrolled CRSwNP. J Clin Med.

[9] Cantone E, Iengo M (2016). Effect of sodium hyaluronate added to topical corticosteroids in chronic rhinosinusitis with **nasal** polyposis. Am J Rhinol Allergy.

[10] De Corso E, Pipolo C, Cantone E, Ottaviano G, Gallo S, Canevari FRM, Macchi A, Monti G, Cavaliere C, La Mantia I, Torretta S, Bussu F, Scarano E, Petrone P, Ghidini A, Lucidi D, Garzaro M, Trimarchi M, Seccia V, Passali GC, Salsi D, Cuda D, Pasquini E, Malvezzi L, Settimi S, Paludetti G, Galli J (2022). Survey on use of local and systemic corticosteroids in the management of chronic rhinosinusitis with nasal polyps: identification of unmet clinical needs. J Pers Med.

[11] Alzahrani MA, Aldriweesh BA, Alharbi MA, Alrashidi TN (2020). Reliability of the Arabic Glasgow children's benefit inventory. Saudi Med J.

[12] Redfors YD, Jönsson R, Tideholm B, Finizia C (2019). Psychometric properties of the Swedish version of the Glasgow Benefit Inventory in otosclerosis subjects. Laryngosc Investig Otolaryngol.

[13] Ali MJ, Iram S, Ali MH, Naik MN (2017). Assessing the outcomes of powered endoscopic dacryocystorhinostomy in adults using the lacrimal symptom (Lac-Q) questionnaire. Ophthalmic Plast Reconstr Surg.

[14] Smirnov G, Tuomilehto H, Kokki H, Kemppainen T, Kiviniemi V, Nuutinen J, Kaarniranta K, Seppa J (2010). Symptom score questionnaire for nasolacrimal duct obstruction in adults-a novel tool to assess the outcome after endoscopic dacryocystorhinostomy. Rhinology.

